# Supplementary material for: Combining Classic and Novel Neutrophil-Related Biomarkers to Identify Non-Small-Cell Lung Cancer
Source: Cancers (Basel). 2024 Jan 25;16(3):513. doi: 10.3390/cancers16030513 (PMC10854517; doi:10.3390/cancers16030513)
Supplement: Supplementary file 1 [file cancers-16-00513-s001.zip › cancers-2793734-supplementary.pdf]

Supplementary Tables

Supplementary Table S1. Host characteristics of the study participants in stage 1.

Characteristics	Control (n=6791)	NSCLC (n=3942)			P value*
		Tis (n=450)	Early (n=3376)	Late (n=116)	
Age	35.266 [29.465-46.770]	50.340 [40.345-58.686]	58.215 [48.679-65.804]	62.865 [55.904-68.591]	<0.001
Sex					
Male	3160 (46.53)	326 (72.44)	2197 (65.08)	50 (43.10)	<0.001
Female	3631 (53.47)	124 (27.56)	1179 (34.92)	66 (56.90)	
BMI (kg/m^2)	23.097 [20.942-25.562]	22.788 [21.145-24.825]	23.125 [21.226-25.182]	23.718 [21.476-25.671]	0.192
Smoking status					
Never	4829 (71.11)	389 (86.44)	2621 (77.64)	66 (56.90)	<0.001
Ever	1281 (18.86)	60 (13.33)	746 (22.10)	50 (43.10)	
Missing	681 (10.00)	1 (0.20)	9 (0.30)	0 (0)	
T stage					
T1	-	-	3231 (95.70)	63 (54.31)	-
T2	-	-	139 (4.12)	31 (26.72)	
T3	-	-	6 (0.18)	12 (10.34)	
T4	-	-	-	10 (8.62)	
N stage					
N0	-	-	3323 (98.43)	14 (12.07)	-
N1	-	-	53 (1.57)	7 (6.03)	
N2	-	-	-	95 (81.90)	
M stage					
M0	-	-	3376 (100.00)	92 (79.31)	-
M1	-	-	-	24 (20.69)	

Early stage indicates stage I&II disease, late stage indicates stage III&IV disease, the staging criteria according to NCCN Clinical Practice Guidelines Non-small cell lung cancer v1, 2022.

NSCLC, non-small cell lung cancer; Tis, carcinoma in situ; SD, standard deviation; BMI, body mass index.

*P indicates the difference across controls, Tis, early-stage NSCLC and late-stage NSCLC.

Values are presented as n (%) unless otherwise specified.

Supplementary Table S2. The associations between classic blood neutrophil-related biomarkers and NSCLC risk in stage 1.

Markers	Control (n=6791)	NSCLC (n=3942)	OR (95%CI)[*]	P value[*]	OR (95%CI)[**, a]	P value[**, a]
NLR						
Low	3394 (49.98)	989 (25.09)	1 (ref)	<0.001	1 (ref)	<0.001
High	3397 (50.02)	2953 (74.91)	2.983 (2.737, 3.252)		2.608 (2.333, 2.918)	
NEU						
Low	3368 (49.60)	1416 (35.92)	1 (ref)	<0.001	1 (ref)	<0.001
High	3423 (50.40)	2526 (64.08)	1.755 (1.620, 1.902)		2.222 (1.994, 2.477)	
LYM						
Low	3378 (49.74)	2741 (69.53)	1 (ref)	<0.001	1 (ref)	<0.001
High	3413 (50.26)	1201 (30.47)	0.434 (0.399, 0.471)		0.650 (0.583, 0.724)	

NLR, neutrophil to lymphocyte ratio; NEU, absolute neutrophil counts; LYM, absolute lymphocyte counts; NSCLC, non-small cell lung cancer; OR, odds ratio; CI, confidence interval.

The value of NLR, NEU, and LYM were divided into low and high groups based on the median value in the control group as the cutoff.

[a] Adjusted factors: age+sex+BMI+smoking status.

[*] shows the OR (95% CI) and p values from univariate logistic regression analysis.

[**] shows the OR (95% CI) and p values from multiple logistic regression analysis adjusting for age, sex, BMI, and smoking status.

Values are presented as n (%) unless otherwise specified.

Supplementary Table S3. Host characteristics of the subset of the study participants with measurements of novel blood neutrophil-related biomarkers.

Characteristics		Control (n=66)	NSCLC (n=132)		P value*
			Tis (n=42)	IAC (n=90)	
Age (years) (mean ± SD)		59.80 ± 8.89	53.33 ± 7.36	62.93 ± 8.73	0.956
BMI (kg/m^2) (mean ± SD)		24.05 ± 2.60	23.21 ± 2.65	23.44 ± 2.86	0.161
Sex					
	Male	32 (48.48)	20 (47.62)	44 (48.89)	0.991
	Female	34 (51.52)	22 (52.38)	46 (51.11)	
Smoking status					
	Never	46 (69.70)	32 (76.19)	53 (58.89)	0.112
	Ever	20 (30.30)	10 (23.81)	37 (41.11)	
Hypertension					
	No	41 (62.12)	34 (80.95)	53 (58.89)	0.041
	Yes	25 (37.88)	8 (19.05)	37 (41.11)	
Diabetes					
	No	57 (86.36)	39 (92.86)	85 (94.44)	0.191
	Yes	9 (13.64)	3 (7.14)	5 (5.56)	
Stage					
	Early (I+II)	-	-	55 (61.11)	-
	Late (III+IV)	-	-	35 (38.89)	
T stage		-			
	T1	-	-	51 (56.67)	
	T2	-	-	36 (40.00)	-
	T3	-	-	2 (2.22)	
	T4	-	-	1 (1.11)	
N stage					
	N0	-	-	44 (48.89)	
	N1	-	-	15 (16.67)	-
	N2			31 (34.44)	
M stage					
	M0	-	-	86 (95.56)	-
	M1	-	-	4 (4.44)	

Tis, carcinoma in situ; IAC, invasive adenocarcinoma; NSCLC, non-small cell lung cancer. Early stage indicates stage I&II disease, late stage indicates stage III&IV disease, the staging criteria according to NCCN Clinical Practice Guidelines Non-small cell lung cancer v1, 2022.

* P indicates the difference across control, Tis and IAC.

Values are presented as n (%) unless otherwise specified.

Supplementary Table S4. The difference in novel neutrophil-related biomarkers in stage 2.

Markers	Control (n=66) median [Q1-Q3]	NSCLC (n=132) median [Q1-Q3]	P value
IL-6 (pg/mL)	0.312 [0.244-0.513]	7.460 [3.511-14.592]	<0.001
CXCL2 (pg/mL)	317.40 [213.73-517.95]	378.20 [275.10-517.20]	0.178
IL-1RA (pg/mL)	205.41 [163.72-266.47]	547.20 [361.60-785.10]	<0.001
IL-1α (pg/mL)	2.598 [1.591-4.130]	3.104 [2.118-4.203]	0.313
CXCL5 (pg/mL)	518.60 [378.00-882.10]	642.80 [456.30-833.50]	0.565
NLR	1.684 [1.350-2.249]	2.471 [1.987-3.197]	<0.001
NEU (10^9/L)	3.155 [2.710-3.728]	3.942 [3.287-4.566]	0.001
LYM (10^9/L)	1.815 [1.613-2.163]	1.625 [1.361-1.798]	0.001

NSCLC, non-small cell lung cancer; NLR, neutrophil to lymphocyte ratio; NEU, absolute neutrophil counts; LYM, absolute lymphocyte counts; IL-6, Interleukin 6; IL-1α, Interleukin 1 alpha; IL-1RA, Interleukin-1 receptor antagonist; CXCL2, C-X-C Motif Chemokine Ligand 2; CXCL5, C-X-C Motif Chemokine Ligand 5.

Supplementary Table S5. The comparison of model performance of models with different predictors.

Models	AUC	95% CI	TPR	FPR
Model 1: BMI+smoking status	0.603	0.527, 0.678	0.742	0.606
Model 2: Model 1+NLR+CEA	0.716	0.637, 0.794	0.652	0.288
Model 4: Model 2+IL-6	0.814	0.746, 0.881	0.864	0.394
Model 5: Model 2+IL-1RA	0.807	0.742, 0.872	0.848	0.394
Model 3: Model 2+IL-6+IL-1RA	0.851	0.793, 0.908	0.856	0.333

BMI, body mass index; NLR, neutrophil to lymphocyte ratio; CEA, carcinoembryonic antigen; CI, confidence interval; AUC, area under the receiver operating characteristic curve; IL-6, Interleukin 6; IL-1RA, Interleukin-1 receptor antagonist; TPR, true positive rate; FPR, false positive rate.

Supplementary Figures

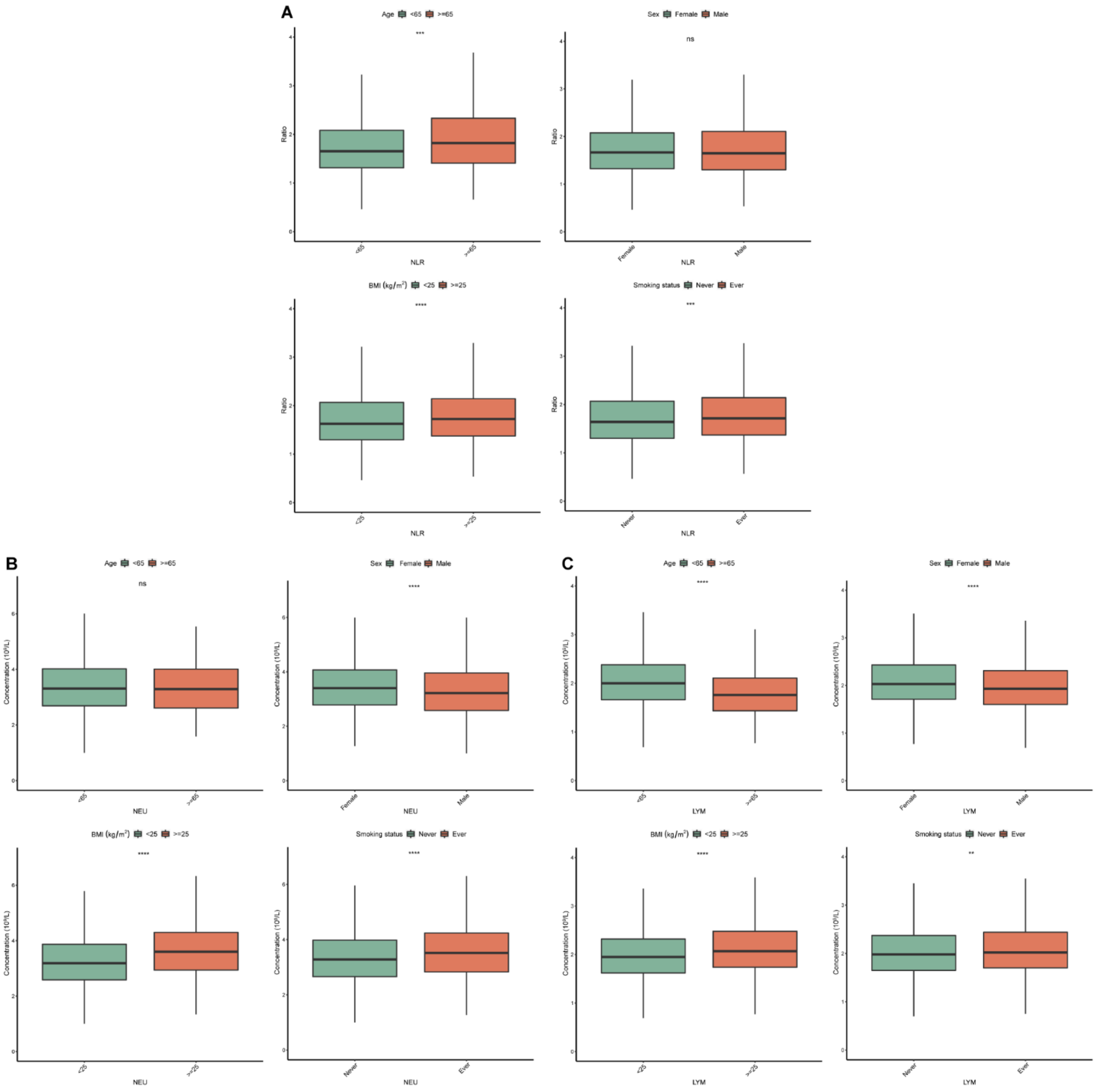

Supplementary Figure S1. The distribution of classic blood biomarkers (NLR, NEU, and LYM) across different epidemiological features (age, sex, BMI, and smoking status) among the healthy controls in stage 1.

(A) The differential analysis of NLR in different groups among age (upper left), sex (upper right), BMI (low left), and smoking status (low right). **(B)** The differential analysis of NEU in different groups among age (upper left), sex (upper right), BMI (low left), and smoking status (low right). **(C)** The differential analysis of LYM in different groups among age (upper left), sex (upper right), BMI (low left), and smoking status (low right). NLR, neutrophil to lymphocyte ratio; NEU, absolute neutrophil counts; LYM, absolute lymphocyte counts; BMI, body mass index. ** $p<0.01$; *** $p<0.001$; **** $p<0.0001$; 'ns' indicates not significant.

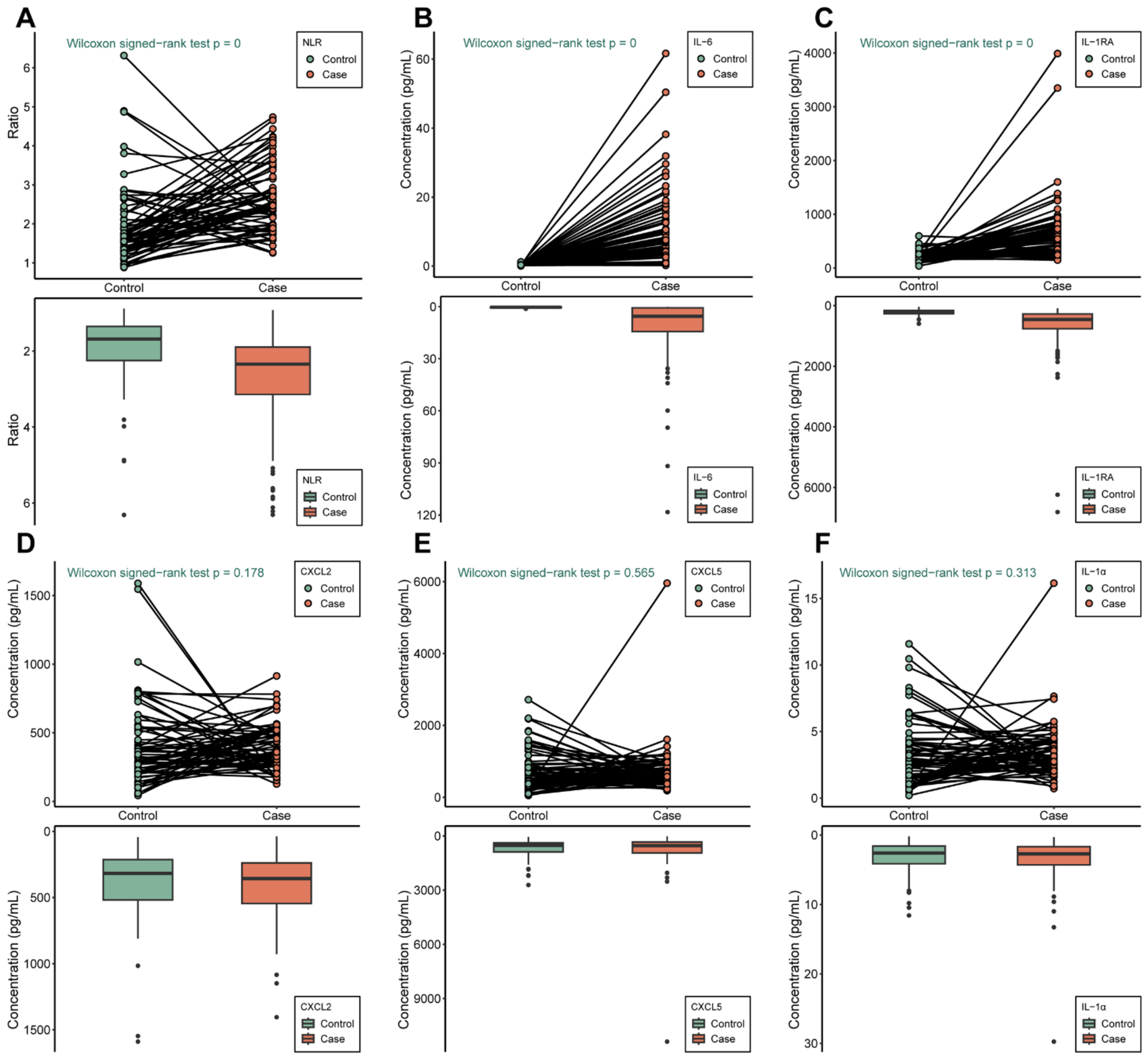

Supplementary Figure S2. The differences analysis of blood neutrophil-related biomarkers in paired samples.

(A) The paired samples' differences analysis of NLR. **(B)** The paired samples' differences analysis of IL-6. **(C)** The paired samples' differences analysis of IL-1RA. **(D)** The paired samples' differences analysis of CXCL2. **(E)** The paired samples' differences analysis of CXCL5. **(F)** The paired samples' differences analysis of IL-1α. NLR, neutrophil to lymphocyte ratio; IL-6, Interleukin 6; IL-1RA, Interleukin-1 receptor antagonist; CXCL2, C-X-C Motif Chemokine Ligand 2; CXCL5, C-X-C Motif Chemokine Ligand 5; IL-1α, Interleukin 1 alpha.

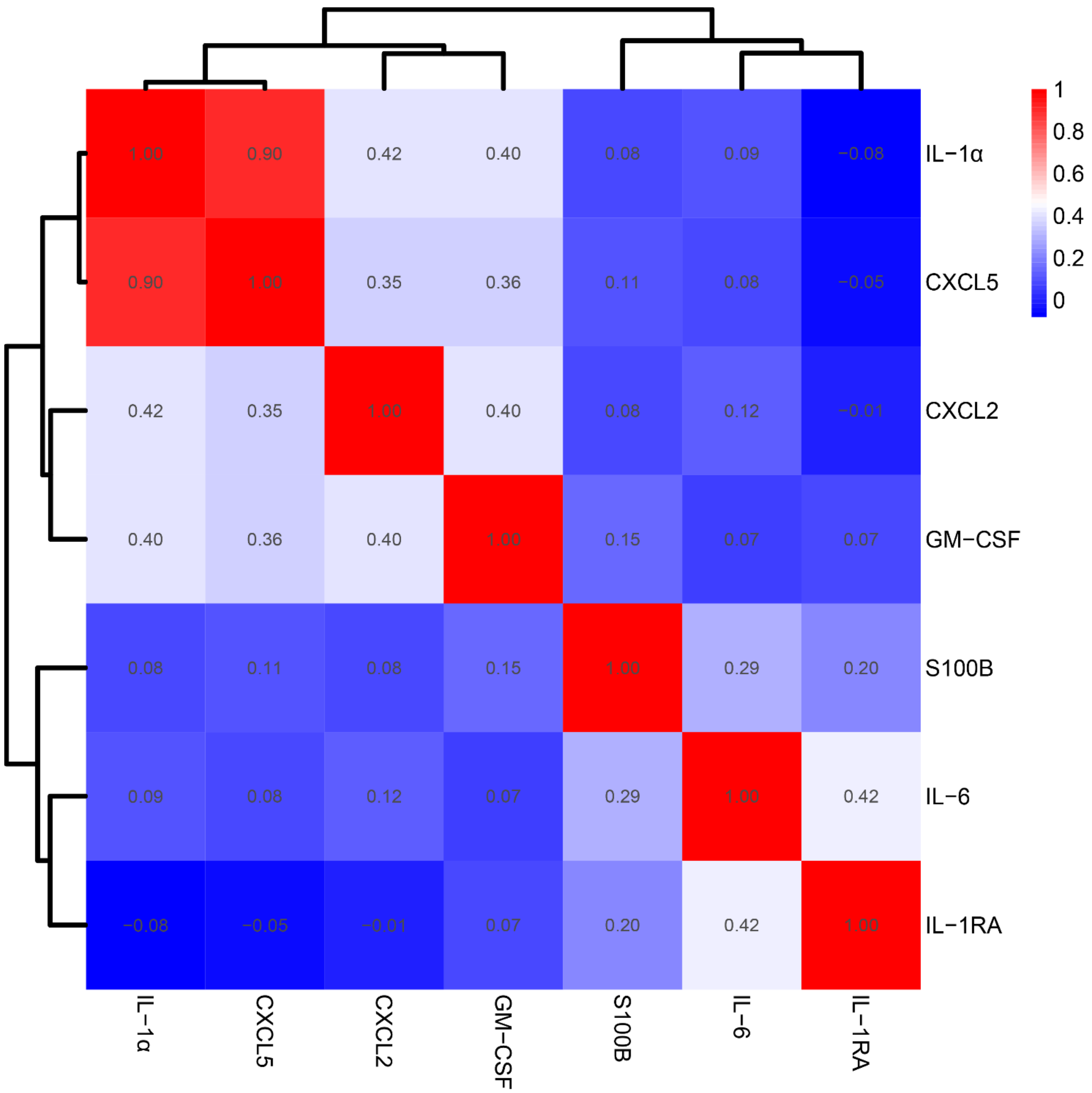

Supplementary Figure S3. The pairwise correlations among the novel blood neutrophil-related biomarkers.

IL-6, Interleukin 6; CXCL2, C-X-C motif chemokine ligand 2; IL-1RA, Interleukin-1 receptor antagonist; IL-1α, Interleukin 1 alpha; CXCL5, C-X-C motif chemokine ligand 5; S100B, S100 calcium binding protein B; GM-CSF, granulocyte-macrophage colony stimulating factor.

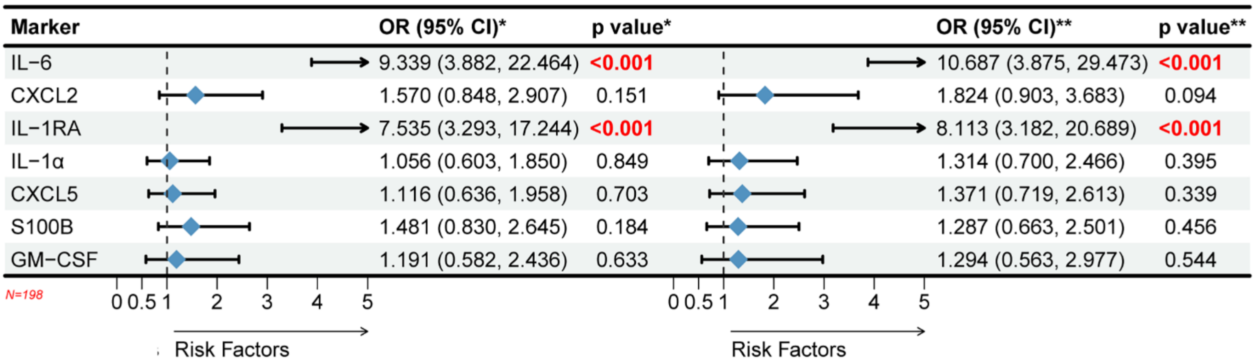

Supplementary Figure S4. The associations between novel blood neutrophil-related biomarkers and NSCLC risk in stage 2.

The univariate logistic regression analysis was shown on the left of figure while the multiple logistic regression analysis on the right. In multiple conditional logistic regression analysis, the factors BMI, smoking status, NLR, and CEA were adjusted. * shows the OR (95%CI) and p values of the univariate conditional logistic regression analysis. ** shows the OR (95%CI) and p values of the multiple conditional logistic regression analysis. NLR, neutrophil to lymphocyte ratio; IL-6, Interleukin 6; CXCL2, C-X-C motif chemokine ligand 2; IL-1RA, Interleukin-1 receptor antagonist; IL-1α, Interleukin 1 alpha; CXCL5, C-X-C motif chemokine ligand 5; S100B, S100 calcium binding protein B; GM-CSF, granulocyte-macrophage colony stimulating factor; OR, odds ratio; CI, confidence interval.
